# Replication Independent Formation of Extrachromosomal Circular DNA in Mammalian Cell-Free System

**DOI:** 10.1371/journal.pone.0006126

**Published:** 2009-07-01

**Authors:** Zoya Cohen, Sara Lavi

**Affiliations:** Department of Cell Research and Immunology, Tel Aviv University, Tel Aviv, Israel; Duke University, United States of America

## Abstract

Extrachromosomal circular DNA (eccDNA) is a pool of circular double stranded DNA molecules found in all eukaryotic cells and composed of repeated chromosomal sequences. It was proposed to be involved in genomic instability, aging and alternative telomere lengthening. Our study presents novel mammalian cell-free system for eccDNA generation. Using purified protein extract we show that eccDNA formation does not involve *de-novo* DNA synthesis suggesting that eccDNA is generated through excision of chromosomal sequences. This process is carried out by sequence- independent enzymes as human protein extract can produce mouse- specific eccDNA from high molecular weight mouse DNA, and *vice versa*. EccDNA production does not depend on ATP, requires residual amounts of Mg^2+^ and is enhanced by double strand DNA breaks.

## Introduction

All eukaryotic cells contain extrachromosomal circular DNA (eccDNA) [1], which originates from the cell's genome. The amount of eccDNA varies greatly in different cells, but in mammals elevated level of these molecules is usually associated with genomic instability and cancer[1], [2], [3], [4]. EccDNA is also considered to participate in the aging process, as its level rises during the aging of yeast and mammalian cells [5], [6].

Several studies showed that tandemly repetitive DNA is the most common substrate for eccDNA formation [7], [8], [9], [10], [11], [12]. Moreover, in all studied organisms eccDNA molecules derived from tandem repeats preserve the multimeric structure of the original sequence, being composed of the basic repeat multiplies [6], [8], [10], [11], [12], [13], [14], [15]. These findings implicate that eccDNA formation is an evolutionary conserved process, which depends on sequence homology and, probably, involves homologous recombination (HR) as it was shown in yeasts [16] However, recently we revealed that DNA Ligase IV (DNL4) [17], a major component of non-homologous end joining (NHEJ), is required for this process in mammalian cells [11]. Thus, considering that mechanism for eccDNA generation is most probably conserved, it seems to engage enzymes from different DNA repair pathways.

One of the major questions in eccDNA research is whether its formation occurs through excision of chromosomal sequences or by replication. The outcome of the first process will be loss of genetic material, while the second will lead to DNA amplification. Since both processes play a significant role in genomic instability and cancer, this issue is of the utmost importance.

The current study is aimed to explore the first steps of eccDNA synthesis in mammalian cells. To this end we have constructed a mammalian cell free system. Using this system we show that eccDNA formation from high molecular weight DNA does not depend on new DNA synthesis, indicating that eccDNA is formed through excision. The process of eccDNA production is not sequence-specific, as human protein extract can produce eccDNA from mouse DNA, and *vice versa*. We also show that this process does not depend on energy and requires residual amounts of Mg^2+^. These results are consistent with our previous finding that the process depends on DNL4 [11], which is found in preadenylated form in mammalian cell extracts [18] and is active at low magnesium level [19]. Altogether, the current study suggests that repetitive sequences from internal chromosomal locations are continually lost from the genome, and persist in a circular extrachromosomal form in the cell. It is also possible that tandem repeats may be inserted back into the chromosome into the pericentromeric region or to other chromosomal sites. Such events may play a role in centromeric shrinkage and expansion, premature ageing, genomic instability, appearance of diseases and tumor formation.

## Materials and Methods

### Cell culture

B16-F10 and HEK 293 cells were propagated in Dulbecco modified Eagle medium (Gibco Laboratories) supplemented with 10% fetal calf serum, 1% glutamine and antibiotics. Cells at ∼80–90% confluence were generally used for extract preparation.

### Extract preparation

Protein extract was prepared according to Fairman et al [20] with minor modifications. The cells (∼5×10^8^) were washed twice with PBS and once with cold buffer A (20 mM Hepes-KOH (pH 7.5), 5 mM KCl, 1.5 mM MgCl_2_, 0.1 mM DTT) and resuspended in cold buffer A at 2×10^8^ cells/ml. After 10 min incubation on ice the cells were homogenized, incubated on ice for additional 30 min, and centrifuged at 4000 g, 4°C for 10 min. The supernatant (cytoplasmic extract, containing also nuclear proteins released during the incubation on ice) was dialyzed against 500 volumes of buffer D (25 mM Tris-HCl (pH 7.5), 1 mM EDTA, 0.01% Nonidet P-40, 10% glycerol, 1 mM DTT, 25 mM NaCl, protease inhibitor cocktail) and stored at −70°C until use. The pellet (nuclei) was washed twice with 20 ml cold buffer B (20 mM Tris-HCl (pH 7.5), 0.5 mM MgCl_2_, 0.5 mM KCl, 0.5 mM DTT, 0.5% Tryton-X-100, protease inhibitor cocktail) and resuspended in 2 ml cold buffer C (500 mM NaCl, 2 mM EDTA, 0.5 mM DTT, 10% sucrose, protease inhibitor cocktail), followed by lysis with three freeze-thaw cycles and centrifugation at 4000 g, 4°C for 10 min. After that the supernatant was precipitated with 70% (NH_4_)_2_SO_4_ at 4°C for 20 min, followed by centrifugation at 15000 g, 4°C for 30 min. The pellet was dissolved in 2 ml buffer D and dialyzed against 500 volumes of the same buffer for 18 h. The nuclear protein extract was snap-frozen and stored at −70°C until use.

### DNA preparation

Genomic (high molecular weight) DNA was prepared according to Sambrook et al [21] with modifications. Cells were washed with PBS and suspended in TE at 5×10^7^ cells/ml. The cell suspension was incubated with 10 volumes lysis buffer (10 mM Tris (pH 8.0), 100 mM EDTA, 0.5% SDS, 20 µg/ml RNAse A) at 37°C for 1 h, followed by 2.5 h incubation with 100 µg/ml proteinase K. After that the lysate was extracted with phenol-chlorophorm, followed by addition of ethanol to 50% and DNA spooling. The spooled DNA was washed three times in 50% ethanol, dried and suspended in H_2_O (final concentration 500 ng/µl).

### *In vitro* eccDNA formation

Genomic DNA (2 µg) was incubated with 0.5 µg nuclear or cytosolic protein extract in reaction buffer containing 30 mM Hepes (pH 7.5), 0.5 mM DTT and other supplies as noted in the figure legends. All reactions were incubated for 24 h, followed by treatment with 20 µg proteinase K at 65°C for 10 min. Same reaction performed with heat inactivated (65°C for 10 min) extract served as negative control (called “Input”) in all experiments.

### Neutral-neutral 2D gel electrophoresis

**Figure 1:**
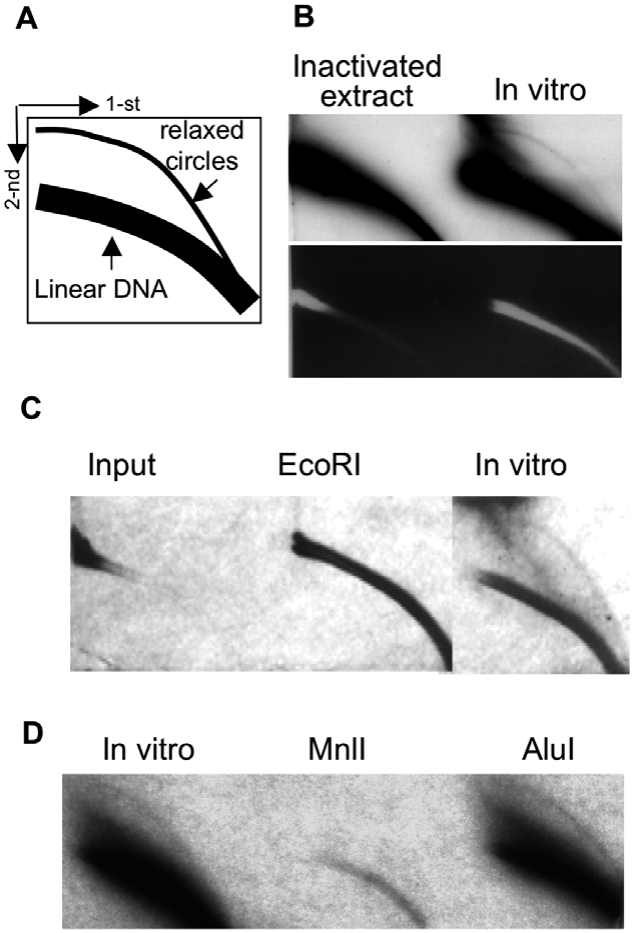
EccDNA formation *in vitro*. A) A scheme of two-dimensional gel electrophoresis (2D gel) showing the migration of linear double strand DNA and relaxed circles. B) Formation of eccDNA from mouse genomic DNA *in vitro* by mouse nuclear protein extract in the presence of 7 mM MgCl_2_, energy-regenerating system (40 mM phosphocreatinine, 10 µg creatine phosphokinase), 4 mM ATP, 250 µM NTPs and 0.5 mM dNTPs (top- hybridization to MSD; bottom- EtBr staining). Note that in addition to eccDNA formation seen upon hybridization, the extract activity caused DNA degradation and thus changed the pattern of linear DNA arc, as is seen from EtBr staining. C). EccDNA is generated in the *in vitro* reaction and is not liberated from HMW input DNA. Mouse DNA was either digested by *EcoRI* or subjected to *in vitro* reaction for eccDNA formation. The blot was hybridized to MSD D). EccDNA produced *in vitro* consist of major satellite DNA. Products of *in vitro* reaction (performed similarly to B) were digested either with *MnlI*, which has 3 recognition sites in MSD or with *AluI*, which does not cut MSD. The blot was hybridized to total mouse genomic DNA.

The products of reactions were separated on two-dimensional (2D) gel as described by Cohen and Lavi [2] (a diagram of 2D gel is shown in Fig. 1a). The gel was blotted onto a Byodine B nylon membrane (Pall Corporation, USA) and hybridized with probes, as noted in the legends.

### Probes

Total human DNA was prepared from HEK 293 cells as described by Sambrook *et al* [21] and used for detection of human DNA. Major satellite DNA (MSD) was prepared as described previously [11] and used for detection of mouse DNA. All probes were labeled either with 50 µCi [α-^32^P]dCTP using a Rediprime II labeling kit (Amersham Biosciences, USA) or with DIG labeling system (Roche). Kodak BMS film was exposed to the membranes or they were quantitatively analyzed using a Fuji BAS1000 PhosphorImager and the Tina 2.07 program (Dinko and Renium, Israel).

### Blot quantification

To prevent the possible effect of unequal transfer on the results, the eccDNA signal was divided by the corresponding linear DNA signal. The signals of the eccDNA arc and of linear DNA arc were calculated by measuring the respective signal and subtracting the background (equal clear area). Since the hybridization conditions may slightly vary between experiments, for quantification of the mean value for several independent experiments, the control value of each experiment was considered 100% and the experimental values were calculated accordingly.

## Results

The investigation of eccDNA phenomenon is problematical due to high heterogeneity of these molecules and lack of adequate methods for its purification from linear DNA. This problem is mainly solved by the use of two-dimensional gel electrophoresis (2D gel) [2], which allows for separation of DNA molecules according to their size and structure. Each DNA population (supercoiled, open circular, linear single- and double stranded), consisting of molecules of heterogeneous size, migrates as a separate arc, allowing simultaneous analysis of size range, amount and sequence content of both supercoiled and open circular eccDNA. Interestingly, the majority of eccDNA is found in open circular conformation. Consistent with this data, all eccDNA in the current study consists of open circular molecules (see scheme in Fig. 1A).

### Formation of eccDNA catalyzed by nuclear protein extract from mammalian cells

To promote the investigation of eccDNA phenomenon in mammalian cells we established a cell-free system for eccDNA formation. We used mouse DNA as a template through this study since mouse genome contains high proportion of major satellite DNA, which was previously shown to be prone to eccDNA formation *in vivo* [11]. Initially the *in vitro* reaction was performed in the presence of Mg^2+^, ATP, energy-regenerating system, NTPs and dNTPs. Nuclear protein extract, purified according to Fairman *et al* [20] was found to be optimal for eccDNA production under these conditions (Fig. 1B). The eccDNA-generating activity was readily eliminated by heat inactivation of the extract, confirming that it depends on the purified proteins. It should be noted that after optimization of reaction conditions cytosolic extract, prepared according to Fairman *et al* [20] provided satisfactory results and was used instead of nuclear due to easier purification procedure (see below).

To exclude the possibility that eccDNA is already present in the input DNA and the extract activity releases it form high molecular weight DNA, the input DNA was digested with *EcoRI*, which does not have recognition sites in major satellite DNA. As expected, the input DNA was found to be free from eccDNA (Fig. 1C).

To examine whether satellite DNA serves as preferential template for eccDNA formation, similarly to the phenomenon observed *in vivo*, the reaction products were digested either with *MnlI*, which has 3 recognition sites in MSD repeat or with AluI that does not cut this sequence. As expected, while the first enzyme completely wiped the eccDNA arc, it was resistant to the second enzyme, as is shown by hybridization to the total DNA (Fig. 1D).

Thus, the cell-free system imitates the process observed *in vivo*, producing eccDNA *de novo* from the input DNA.

### Enzymes engaged in eccDNA production are not sequence- specific

**Figure 2:**
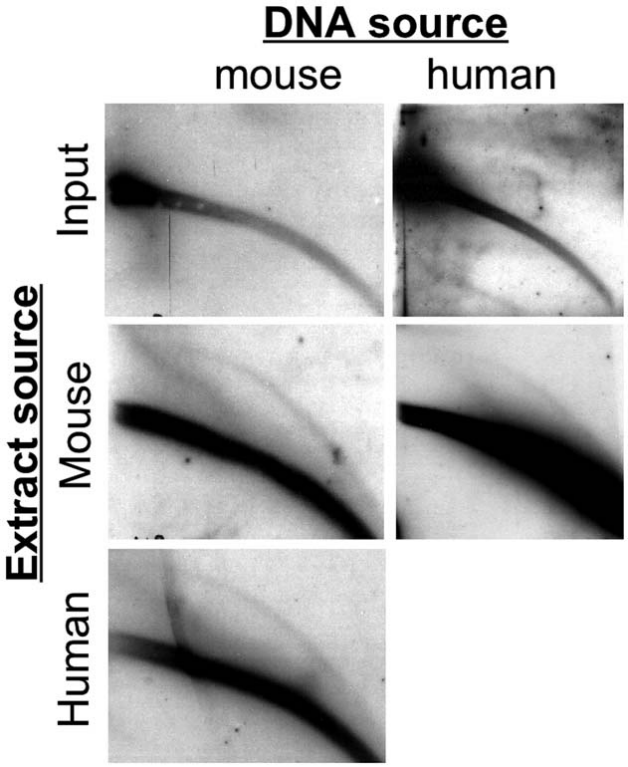
EccDNA – generating enzymes are not sequence-specific. Human genomic DNA was incubated with either heat-inactivated or native mouse nuclear protein extract. The samples were separated on 2D gel, blotted and hybridized to total human DNA probe. Mouse genomic DNA was incubated with either mouse (inactivated or native) or human nuclear protein extract, under the conditions described in Fig.1B. The samples were separated on 2D gel, blotted and hybridized to MSD probe.

Earlier studies showed that tandemly repetitive DNA is the preferential substrate for eccDNA formation [7], [8], [9], [10], [11], [12]. Such tendency can evolve either from structural characteristics of repetitive DNA or from sequence-specific enzymes. Studies with Xenopus egg extracts demonstrated that eccDNA can be formed from virtually any repetitive DNA [8], arguing in favor of the first possibility. To test whether the same is true in mammalian system, we examined the ability of human nuclear extract to produce eccDNA from mouse DNA and *vice versa*. Since repetitive sequences of these organisms differ greatly, positive results would mean that eccDNA formation does not depend on sequence-specific enzymes. Indeed, as shown in Figure 2, mouse extract produced eccDNA form human DNA. Consistent with that, human extract produced eccDNA harboring mouse MSD from mouse genomic DNA with the same efficiency as the mouse extract. As MSD is specific to mouse and no homologous sequences are found in human genome, eccDNA in mammalian cells is almost certainly formed by non-sequence specific enzymes.

### EccDNA formation depends on trace amounts of magnesium

**Figure 3:**
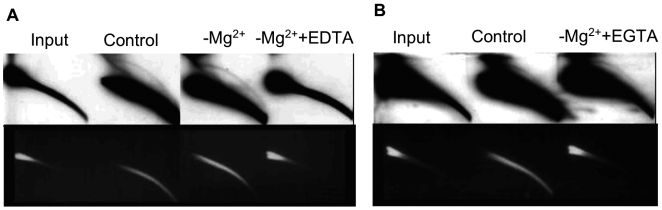
Residual level of magnesium is sufficient for eccDNA formation. A) eccDNA formation depends on trace amounts of ions present in protein/DNA preparations. Mouse DNA was incubated with mouse nuclear protein extract under conditions described in 1B either in the presence or absence of Mg^2+^ and 25 mM EDTA. Top- hybridization, bottom- EtBr staining. B) Chelation with EGTA does not affect the reaction. The reactions were performed similarly to (A) in the absence or presence of 25 mM EGTA. Top- hybridization, bottom- EtBr staining. All blots were hybridized to MSD probe.

Most of the enzymes, directly involved in DNA metabolism, require divalent ions for their activity. DNL4, which is engaged in eccDNA production, differs from other DNA ligases in its ability to act at very low concentration of magnesium [19]. To examine the dependence of eccDNA formation on Mg^2+^, we performed the *in vitro* reaction in the absence of this ion. Surprisingly, the signal of eccDNA arc was the same or even stronger than the one in the control reaction (Fig. 3A). In addition, the degradation of linear DNA, observed in the control reaction (and in most of the reactions through the article) was much less prominent in the absence of Mg^2+^. However, addition of EDTA completely blocked both eccDNA formation and linear DNA degradation, suggesting that the reaction contains traces of divalent ions, which are sufficient for both processes to occur. The origin of these ions is probably in the protein extract, as metal-containing buffers are utilized during its preparation. To better specify the requirements for divalent ions, we carried out the same experiment in the presence of EGTA. The major difference between these chelators is that EDTA has strong affinity for magnesium, while EGTA binds magnesium very weakly. As seen in Figure 3B, linear DNA degradation was totally abrogated by this treatment (EtBr staining), similarly to the effect of EDTA, but eccDNA formation was not affected (hybridization). These results indicate that eccDNA formation depends on magnesium; however, residual amounts of this ion are sufficient for the process. It is consistent with the involvement of DNL4, which is active at low magnesium level [19] and suggests that same conditions are sufficient for other enzymes involved in eccDNA generation. The fact that linear DNA degradation was prevented by both EDTA and EGTA is coherent with the fact that the majority of DNA nucleases utilize Zn^2+^, which is eliminated by both chelators.

### EccDNA production *in vitro* is energy-independent

**Figure 4:**
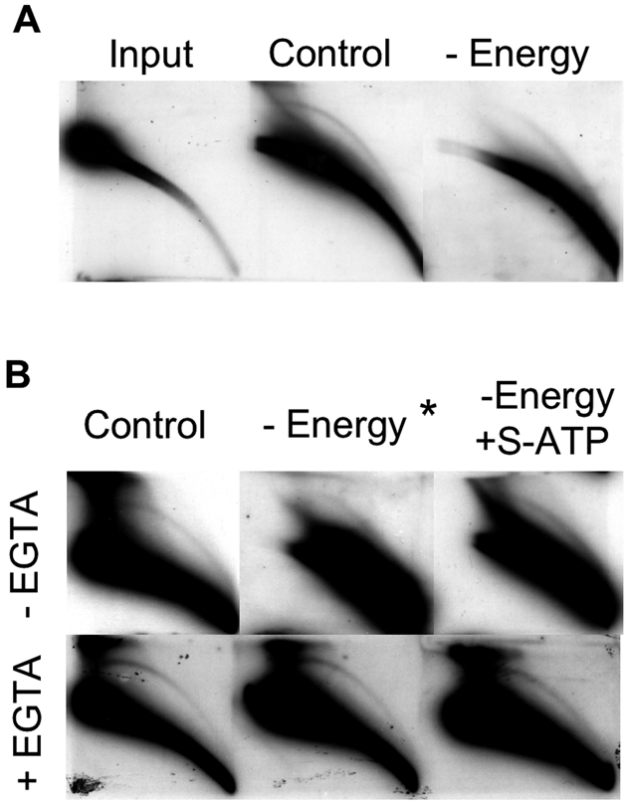
The reaction does not require energy supplement. A) Formation of eccDNA in the absence of energy input. Mouse DNA was incubated with mouse nuclear protein extract under the conditions described in 1B, either in the presence or absence of energy regenerating system and ATP. B) Effect of γ-S-ATP on the reaction. The reactions were performed similarly to (A) in the absence or presence of 50 µg/ml γ-S-ATP and EGTA. The blots were hybridized to MSD probe.

Previously we suggested that eccDNA synthesis occurs in two steps: generation of linear multimers and their ligation into circles by DNL4 [17]. DNL4 activity is independent of ATP *in vitro* since the enzyme is found in preadenylated form in cell extracts [18]. Hence, characterization of the energy requirements for eccDNA formation from genomic DNA could narrow down the list of candidate enzymes engaged in the first steps of the process. To address this question we performed the reaction in the absence of energy (energy regenerating system and ATP). Surprisingly, the production of eccDNA did not stop in the absence of energy source, although the amount of large eccDNA molecules was significantly reduced (Fig. 4A). However, the smaller eccDNA molecules were generated with same intensity as in the control reaction. This change in size range correlated with massive degradation of linear DNA, suggesting that the reduced amount of large eccDNA in the absence of energy resulted from the reduced size of available DNA template. To exclude the possibility of ATP contaminants in the reaction (as was shown for magnesium in previous section), we utilized non-hydrolysable ATP analog, γ-S-ATP. As seen from Figure 4B (top), this treatment did not prevent eccDNA formation. On the contrary, γ-S-ATP slowed down the degradation of linear DNA (probably, by inhibiting ATP-dependent nucleases), observed in the reaction without energy supplement, and consequently, enhanced eccDNA formation. This result demonstrates that the reduced eccDNA formation in the absence of energy results most likely from intensive degradation of linear DNA, which serves as a source of eccDNA and implicates that that neither step of eccDNA formation consumes energy. To prove this statement we exploited EGTA to prevent template DNA degradation in energy-depleted reactions. As expected, this treatment abrogated fragmentation of linear DNA and resulted in similar levels of eccDNA in control, energy-free and ATP-depleted reactions (Figure 4B, bottom). Thus, eccDNA formation does not require the addition of ATP.

### EccDNA formation does not require DNA synthesis

**Figure 5:**
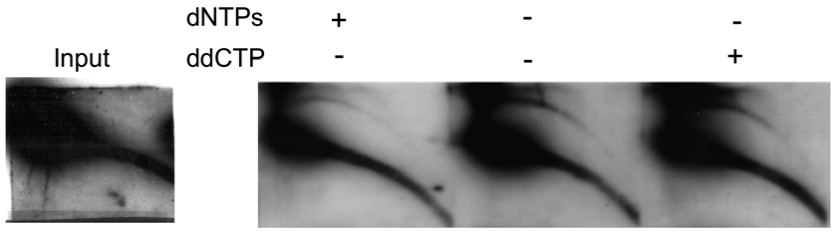
eccDNA formation does not require new DNA synthesis. Mouse DNA was incubated with mouse nuclear protein extract under the conditions described in Materials and Methods, supplied with 25 mM EGTA and either in the presence or absence of 500 µM dNTPs, 250 µM NTPs and 500 µM ddCTP. Blots were hybridized to MSD probe.

There are two major theories regarding the origin of eccDNA: aberrant replication of genomic DNA followed by formation of eccDNA from extra copies of genomic material, and excision of chromosomal DNA with subsequent ligation of the excised fragments. To address this question we examined the requirement for DNA synthesis precursors, dNTPs. As seen in Figure 5, the absence of dNTPs did not affect eccDNA production, suggesting that they are formed by excision from chromosomal DNA. Since the reaction contains traces of small molecules, we wished to exclude the possibility that eccDNA formation resulted from traces of dNTPs. To this end the reaction was performed in absence of dNTPs and in the presence of dideoxycitidine, which is known inhibitor of DNA synthesis. As shown in Figure 5, this treatment had no effect on eccDNA generation, confirming that it does not depend on new DNA synthesis. Thus, eccDNA is most likely generated through an excision of chromosomal sequences.

### Formation of eccDNA by cytosolic extract

**Figure 6:**
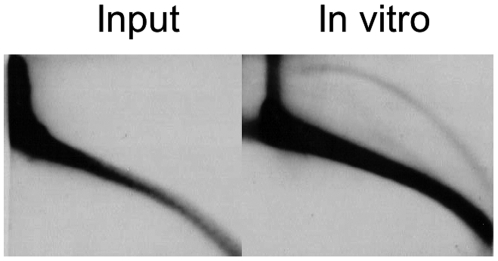
Formation of eccDNA *in vitro* by cytosolic extract. Mouse genomic DNA was incubated with Hela cytosolic extract under conditions described in Materials and Methods and the presence of 25 mM EGTA without additional supplies. The blot was hybridized to MSD.

Nuclear extract utilized for *in vitro* eccDNA production through the study requires long purification procedure. Thus, after optimization of reaction conditions we tried to use cytosolic extract [20], which contains nuclear proteins released during incubation in hypotonic buffer. As seen in Fig. 6, the cytosolic extract caused efficient eccDNA formation from genomic DNA, and, thus, can be used for the *in vitro* system instead of the nuclear extract.

### Formation of eccDNA from an artificial substrate

**Figure 7:**
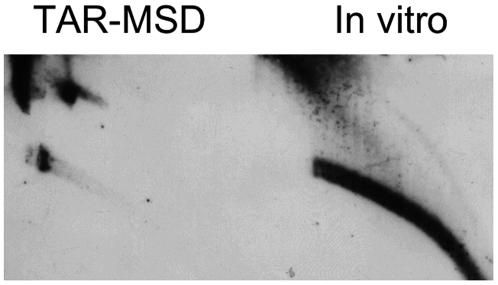
Formation of eccDNA *in vitro* from an artificial substrate. TAR vector containing ∼35 kb MSD insert was incubated with HeLa cytosolic extract under conditions described in Materials and Methods in the presence of 25 mM EGTA without additional supplies. The blot was hybridized to MSD.

To further ascertain the formation of eccDNA *in vitro* we performed the reaction using artificial substrate as a template DNA. TAR vector containing ∼35 kb insert of mouse major satellite DNA, kindly provided by Larionov [22] was used as a substrate. As shown in Fig. 7, the vector served as an adequate template and allowed the generation of eccDNA under the optimized conditions, e.g. without addition of energy source and magnesium and in the presence of EGTA.

### EccDNA is elevated upon induction of DNA double-strand breaks

**Figure 8:**
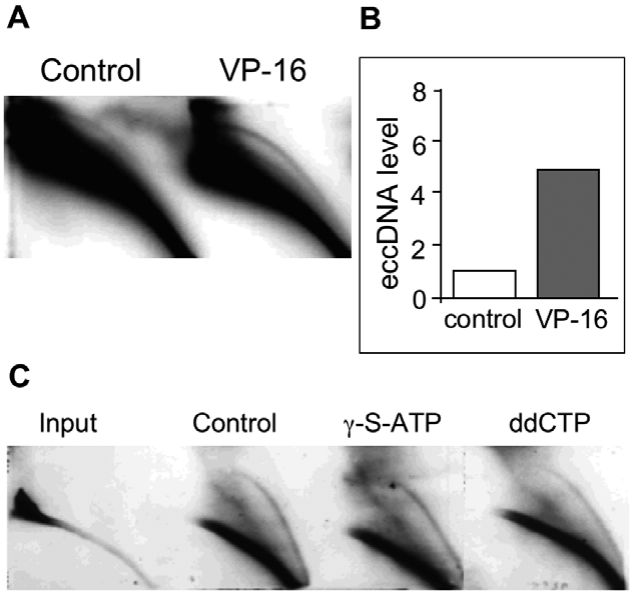
eccDNA generation is enhanced in the presence of DNA-damaging agents. A) Mouse DNA was incubated with mouse nuclear protein extract under conditions described in Materials and Methods in the presence of 25 mM EGTA and either in absence or presence of 2.5 mg/ml VP-16 (etoposide) B) Quantitative analysis of the result presented in (A), based on 4 independent experiments. All blots were hybridized to MSD probe. C) Formation of eccDNA upon induction of DSB does not require energy and is independent of DNA synthesis. Mouse DNA was incubated with HeLa cytosolic protein extract in the presence of 25 mM EGTA, 2.5 mg/ml VP-16 (etoposide) in the presence or absence of 500 µM ddCTP or 50 µg/ml γ-S-ATP as noted.

A variety of DNA damaging agents, including hydroxyurea, MNNG, MMS, and so on were shown to increase eccDNA synthesis *in vivo* [1], [2], [3], [5], [10]. Although the mode of action of these agents differs, the eventual result is generally generation of DNA breaks. However, in the cell's context it is impossible to distinguish between the direct contribution of a DSB to eccDNA elevation and the indirect effect resulting from replication fork blockage, cell cycle arrest and change in protein expression. Therefore, we wished to exploit the cell free system, in which the above obstacles do not affect eccDNA formation. To this end we used a known DNA damaging agent etoposide (VP-16), which causes DSB through the inhibition of topoisomerase II ligase activity [23]. As seen in Figure 8 A and B, VP-16 caused ∼4 –fold elevation in eccDNA level. Similarly to results shown above the formation of eccDNA in the presence of VP-16 was independent of energy and did not involve new DNA synthesis (Fig. 8 C). Thus, DSB probably initiates processes leading to eccDNA formation in cell.

## Discussion

Here we show for the first time the formation of extrachromosomal circular DNA from genomic DNA by an activity present in nuclear protein extract, purified from mammalian cells. Using this system we determined the chemical requirements of the process and demonstrated that eccDNA is formed by excision from the chromosome, thereby leading to a loss of genomic sequences. The eccDNA-generating activity probably includes several proteins, one of which is DNL4 [11]. The other enzymes, which are still to be discovered, should be engaged in the excision of chromosomal fragments and, probably, in recruitment of the DNL4 to the site. Elevated production of eccDNA upon induction of DSB suggests that this event may initiate eccDNA formation under physiological conditions.

Since the majority of eccDNA in all studied organisms consists of tandemly repetitive sequences and appears as perfect multimers of the basic repeat unit [7], [8], [9], [10], [11], [12], its formation most probably involves sequence alignment. As the process does not requires new DNA synthesis the most logical model is that DSB activates mechanism(s) that under certain conditions may lead to the excision of repetitive DNA fragments from the chromosome and their ligation to circular DNA molecules.

### Optimal conditions for eccDNA formation

According to the presented data, eccDNA formation depends on magnesium, but trace amount of this ion is sufficient for the reaction to occur. This condition fits our previous findings on DNL4 involvement [11], [19] and suggests further that additional enzymes, engaged in the process, either do not require ions for their activity or have requirements similar to DNL4 in this regard.

Similarly, the fact that eccDNA is formed in the absence of energy input correlates with DNL4 involvement [18] and restricts the list of possible candidates for the first stages of the process. The perspective enzymes have either to be energy-independent or to persist in activated form in cell extracts, as does DNL4.

In our previous study [11] we have shown that eccDNA formation is elevated in dividing cells. Since we show that eccDNA formation does not require DNA replication, our early findings probably reflect the fact that some of the eccDNA – generating enzymes are cell-cycle – dependent and are active at the S/G2 phase(s).

Although our findings do not determine the enzymes involved in the studied phenomenon, their features are characterized and thereby open further research directions. The ability of extracts to produce eccDNA from non-self DNA excludes all the sequence-specific enzymes and indicates that the process is guided by the repetitive nature of the sequence rather then by sequence recognition.

### Formation of eccDNA through a loss of genomic sequences

For a long time the question of eccDNA origin remained open, and most of the proposed models for its formation included new DNA synthesis to different extents. These models contradicted the data demonstrated by Smith and Vinograd [24] that in cycloheximide-arrested human cells eccDNA consists of pre-existing DNA. Moreover, Cohen *et al* [9] showed that aphidicolin does not prevent the formation of eccDNA in Xenopus egg extracts. Consistent with these findings, we provide a direct evidence for eccDNA formation in conditions that do not permit new DNA synthesis (in the absence of dNTPs). Moreover, addition of ddCTP to inhibit incorporation of dNTPs, which could contaminate the reaction, did not alter eccDNA production. Thus, eccDNA can be formed by excision of repetitive sequences from chromosomal DNA, thereby leading to the loss of these fragments from the genome. Yet, these sequences are preserved during evolution, probably due to repeat expansion processes [25].

The fact that DNA loss occurs mainly at repetitive non-coding sequences does not lessen its significance. On the contrary, it is possible that the tandem repeats at the centromeric region act as *timekeeper* and/or *stresskeeper* and that similarly to the events taking place at telomeres, deletion of these repeats from the centromere region may be hazardous to the cells and might lead to premature aging, genomic instability and initiation of carcinogenesis.
